# Decision criteria for MALDI-TOF MS-based identification of filamentous fungi using commercial and in-house reference databases

**DOI:** 10.1186/s12866-017-0937-2

**Published:** 2017-01-31

**Authors:** Anne-Cécile Normand, Carole Cassagne, Magali Gautier, Pierre Becker, Stéphane Ranque, Marijke Hendrickx, Renaud Piarroux

**Affiliations:** 1 Laboratoire de Parasitologie-Mycologie, CHU Timone, Université de la Méditerranée, Marseille, France; 2 Service of Mycology and Aerobiology, BCCM/IHEM Fungal Collection, Scientific Institute of Public Health, Brussels, Belgium

**Keywords:** Mass Spectrometry, Matrix-Assisted Laser Desorption/Ionization, Filamentous Fungi, Interpretation criteria, log(score) threshold

## Abstract

**Background:**

Several Matrix-Assisted Laser Desorption/Ionization Time-of-Flight mass spectrometry protocols, which differ in identification criteria, have been developed for mold and dermatophyte identification. Currently, the most widely used approach is Bruker technology, although no consensus concerning the log(score) threshold has been established. Furthermore, it remains unknown how far increasing the number of spots to compare results might improve identification performance.

In this study, we used in-house and Bruker reference databases as well as a panel of 422 isolates belonging to 126 species to test various thresholds. Ten distinct identification algorithms requiring one to four spots were tested.

**Results:**

Our findings indicate that optimal results were obtained by applying a decisional algorithm in which only the highest score of four spots was taken into account with a 1.7 log(score) threshold. Testing the entire panel enabled identification of 87.41% (in-house database) and 35.15% (Bruker database) of isolates, with a positive predictive value (PPV) of 1 at the genus level for both databases as well as 0.89 PPV (in-house database) and 0.72 PPV (Bruker database) at the species level. Applying the same rules to the isolates for which the species were represented by at least three strains in the database enabled identification of 92.1% (in-house database) and 46.6% (Bruker database) of isolates, with 1 PPV at the genus level for both databases as well as 0.95 PPV (in-house database) and 0.93 PPV (Bruker database) at the species level.

**Conclusions:**

Depositing four spots per extract and lowering the threshold to 1.7, a threshold which is notably lower than that recommended for bacterial identification, decreased the number of unidentified specimens without altering the reliability of the accepted results. Nevertheless, regardless of the criteria used for mold and dermatophyte identification, commercial databases require optimization.

**Electronic supplementary material:**

The online version of this article (doi:10.1186/s12866-017-0937-2) contains supplementary material, which is available to authorized users.

## Background

Until a few years ago, medical laboratories performed mold identification using phenotypic characterization of colonies and occasional sequencing of informative DNA targets. Currently, the MALDI-TOF MS (Matrix-Assisted Laser Desorption/Ionization Time-of-Flight Mass Spectrometry) approach appears to be a promising method to identify molds and dermatophytes [1–8].

Many teams around the world have demonstrated that filamentous fungi could be identified by using MALDI-TOF MS. They first focused attention on a few specific mold and dermatophyte genera [1, 9–16]. Only after 2010 did research teams begin optimizing protocols to establish a MALDI-TOF MS-based identification approach for routine diagnosis of filamentous fungi and dermatophytes [2–7, 17–21]. Currently, four commercial systems are available for routine diagnosis, and an increasing number of publications have tested their performance in identifying molds and dermatophytes. Some teams have assessed the Saramis [12, 18, 22, 23], Vitek MS [24–28] or Andromas [29] systems; however, most publications refer to the Bruker technique [1–8, 16, 17, 19–21, 26, 30, 31]. According to the Bruker recommendations, the level of similarity between an unknown tested specimen and a reference sample is indicated by a log(score), which is referred to herein as “score”. A score > 2.3 indicates “highly probable species identification”, a score > 2 and < 2.299 indicates “secure genus identification, probable species identification”, a score > 1.7 and < 1.999 indicates “probable genus identification”, and a score < 1.7 indicates “unreliable identification”.

However, these score thresholds have been designed for bacteria identification and are not necessarily appropriate for fungi (especially molds and dermatophytes) while using the manufacturer’s reference database [4, 7, 17, 32, 33]. The majority of publications dealing with MALDI-TOF MS routine identification of filamentous fungi have used a single score threshold, usually ranging from 1.7 to 2.0, depending on the study [3, 4, 6, 8, 17]. However, a study by Shultness et al. [8] has compared the 1.7 and 2.0 score thresholds for MALDI-TOF MS identification after a liquid culture growth step in which no assessment was performed to justify these thresholds. Additionally, while the manufacturer typically recommends a single spot for bacterial identification, some authors have advocated the use of several spots of the same sample to identify molds and dermatophytes [2, 21]. According to these authors, the concordance between identification results can be tested using replicates. Theoretically, this may improve the reliability of identification results, although no study has thoroughly evaluated the optimal number of spots and log(score) threshold for mold identification. Altogether, the use of various thresholds and validation criteria for mold and dermatophyte identification complicate the interpretation of results.

The present study assessed 10 distinct identification algorithms combining several thresholds for one to four spots per sample. Because the Bruker reference database is not comprehensive enough to enable proper identification of many mold species, [4, 8] identification algorithms were assessed using an extensive in-house database (5044 references belonging to 619 species and 165 genera) and the Bruker commercial database (V3.2.1.1 : 604 references belonging to 237 species and 67 genera). Our objective was to optimize the fungal identification workflow in laboratories equipped with a Bruker MALDI-TOF MS identification platform.

## Methods

### Reference databases

The in-house reference database was constructed using a Microflex LT system (Bruker Daltonics, Bremen, Germany) in coordination with the BCCM/IHEM culture collection and the Mycology Laboratory of the AP-HM with the protocol described by Normand et al. [6] (several strains per species, four subcultures per strain and 10 spots per subculture, for a total of four references per strain). Details of the MS in-house database are listed in Additional file 1: Table S1.

### Assessment panel

The assessment panel consisted of filamentous fungus strains that were identified via both DNA sequencing and MALDI-TOF MS. The strains were either selected at the Mycology Laboratory of Marseille (*n* = 224) or the BCCM/IHEM in Brussels (*n* = 198). Of the 224 strains from Marseille, 177 were included because the identification at the species level had to be either clarified or confirmed at the time of the study, while the remaining 47 were randomly selected among frequently identified species in the clinical laboratory. The 198 Belgian strains either originated from the BCCM/IHEM collection or were clinical strains from a previous study [34]. The 422-strain panel included 126 different species belonging to 38 genera, of which 280 strains were represented three or more times in the MALDI-TOF MS reference database (191 strains for the commercial Bruker database), 130 strains were represented once or twice (76 strains for the commercial Bruker database), and 12 strains were not represented in the reference database (155 strains for the commercial Bruker database) (Tables 1 and 2). All samples were first included in the study panel, even those corresponding to species absent from the databases to ensure that the tested algorithms correctly excluded absent species. Next, the identification process was tested again, this time only including the isolates corresponding to species represented by at least three distinct strains in the database to comply with the recommendations we have issued in a previous study [6].

**Table 1:** Panel description obtained after DNA sequencing of the strains and representation of the entire set of species in the in-house reference spectrum database

Species not represented in the in-house reference database	TOTAL (*n* = 12)	Collection strains BCCM/IHEM	Clinical strains (Marseille Hospital)	Species represented once or twice in the in-house reference database	TOTAL (*n* = 130)	Collection strains BCCM/IHEM	Clinical strains (Marseille Hospital)	Species represented three or more times in the in-house reference database	TOTAL (*n* = 280)	Collection strains BCCM/IHEM	Clinical strains (Marseille Hospital)
*Cladosporium bruhnei*	1	1	0	*Acremonium breve*	1	0	1	*Alternaria alternata*	2	1	1
*Cladosporium halotolerans*	2	2	0	*Acremonium strictum*	6	6	0	*Aspergillus alabamensis*	4	0	4
*Colletotrichum lineola*	1	1	0	*Alternaria infectoria*	2	1	1	*Aspergillus caesiellus*	1	1	0
*Trichoderma orientale*	1	1	0	*Arthrinium arundinis*	1	0	1	*Aspergillus calidoustus*	10	2	8
*Mucor hiemalis*	1	1	0	*Aspergillus dimorphicus*	1	0	1	*Aspergillus carbonarius*	1	0	1
*Penicillium atramentosum*	1	1	0	*Aspergillus fischeri*	2	0	2	*Aspergillus carneus*	1	0	1
*Penicillium concentricum*	1	1	0	*Aspergillus flavipes*	3	2	1	*Aspergillus chevalieri*	4	2	2
*Phaeosphaeria avenaria*	1	1	0	*Aspergillus floccosus*	3	0	3	*Aspergillus clavatus*	2	2	0
*Pleospora papaveracea*	1	1	0	*Aspergillus fumigatiaffinis*	2	0	2	*Aspergillus creber*	1	1	0
*Trichoderma reesei*	2	2	0	*Aspergillus iizukae*	1	0	1	*Aspergillus flavus*	15	5	10
				*Aspergillus quadrilineatus*	1	0	1	*Aspergillus fumigatus*	20	4	16
				*Aspergillus ruber*	2	2	0	*Aspergillus hiratsukae*	1	0	1
				*Aspergillus sclerotiorum*	3	1	2	*Aspergillus hollandicus*	5	4	1
				*Aspergillus subolivaceus*	1	0	1	*Aspergillus insuetus*	1	0	1
				*Aspergillus unguis*	4	4	0	*Aspergillus nidulans*	8	4	4
				*Aureobasidium pullulans*	3	2	1	*Aspergillus niger*	4	2	2
				*Cladosporium cladosporioides*	1	1	0	*Aspergillus nomius*	1	0	1
				*Cladosporium pseudocladosporioides*	2	2	0	*Aspergillus parasiticus*	2	1	1
				*Cladosporium sphaerospermum*	1	1	0	*Aspergillus sydowii*	6	2	4
				*Cochliobolus hawaiiensis*	2	0	2	*Aspergillus tamarii*	2	2	0
				*Curvularia lunata*	2	2	0	*Aspergillus terreus*	10	3	7
				*Fusarium dimerum*	2	1	1	*Aspergillus tubingensis*	29	1	28
				*Geosmithia argillacea*	1	0	1	*Aspergillus versicolor*	3	2	1
				*Geosmithia pallida*	1	1	0	*Aspergillus westerdijkiae*	1	0	1
				*Hexagonia hydnoides*	1	0	1	*Beauveria bassiana*	3	2	1
				*Lecytophophora sp.*	1	1	0	*Epicoccum nigrum*	2	2	0
				*Macrophomina phaseolina*	1	0	1	*Exophiala dermatitidis*	2	2	0
				*Mucor circinelloides*	5	5	0	*Fomitopsis pinicola*	2	0	2
				*Penicillium asturianum*	1	0	1	*Fusarium equiseti*	1	0	1
				*Penicillium brasilianum*	3	1	2	*Fusarium oxysporum*	21	15	6
				*Penicillium cecidicola*	2	1	1	*Fusarium proliferatum*	10	1	9
				*Penicillium citrinum*	5	4	1	*Fusarium sacchari*	1	0	1
				*Penicillium corylophilum*	4	2	2	*Fusarium solani*	7	6	1
				*Penicillium crateriforme*	2	1	1	*Fusarium verticillioides*	1	1	0
				*Penicillium diversum*	1	1	0	*Galactomyces geotrichum*	2	2	0
				*Penicillium expansum*	2	2	0	*Lichtheimia corymbifera*	2	2	0
				*Penicillium funiculosum*	2	0	2	*Microsporum audouinii*	1	0	1
				*Penicillium glabrum*	9	3	6	*Microsporum canis*	2	0	2
				*Penicillium helicum*	1	0	1	*Purpureocillium lilacinus*	3	2	1
				*Talaromyces marneffei*	1	0	1	*Paecilomyces variotii*	3	2	1
				*Penicillium nalgiovense*	1	0	1	*Penicillium chrysogenum*	27	13	14
				*Penicillium oxalicum*	1	1	0	*Penicillium crustosum*	3	2	1
				*Penicillium pinophilum*	3	0	3	*Penicillium griseofulvum*	1	1	0
				*Penicillium polonicum*	6	6	0	*Rhizomucor pusillus*	2	2	0
				*Penicillium purpurogenum*	1	0	1	*Rhizopus oryzae*	6	5	1
				*Penicillium raistrickii*	1	1	0	*Scedosporium apiospermum*	11	2	9
				*Penicillium rolfsii*	1	0	1	*Scedosporium prolificans*	3	2	1
				*Penicillium rugulosum*	1	1	0	*Schizophyllum commune*	4	1	3
				*Penicillium sizovae*	2	0	2	*Scopulariopsis brevicaulis*	15	13	2
				*Penicillium spinulosum*	2	0	2	*Trichoderma longibrachiatum*	2	2	0
				*Penicillium terrigenum*	1	0	1	*Trichophyton interdigitale*	1	0	1
				*Penicillium variabile*	2	0	2	*Trichophyton rubrum*	8	0	8
				*Phanerochaete chrysosporium*	2	0	2				
				*Pithomyces chartarum*	3	3	0				
				*Pseudallescheria boydii*	1	0	1				
				*Pseudallescheria ellipsoidea*	1	1	0				
				*Rhizomucor variabilis*	2	0	2				
				*Rhizopus microsporus*	1	1	0				
				*Scedosporium aurantiacum*	6	6	0				
				*Tyromyces fissilis*	3	0	3

**Table 2:** Representation of the entire set of species included in the Bruker reference spectrum database

Species not represented in the Bruker reference database	Total (*n* = 155)	List of the 26 species represented once or twice in the Bruker reference database	Total (*n* = 76)	List of the 25 species represented three or more times in the Bruker reference database	Total (*n* = 191)
*Acremonium breve*	1	*Acremonium strictum*	6	*Alternaria alternata*	2
*Alternaria infectoria*	2	*Aspergillus clavatus*	2	*Aspergillus flavus*	15
*Arthrinium arundinis*	1	*Aspergillus hollandicus*	5	*Aspergillus fumigatus*	20
*Aspergillus alabamensis*	4	*Aspergillus sclerotiorum*	3	*Aspergillus nidulans*	8
*Aspergillus caesiellus*	1	*Aspergillus sydowii*	6	*Aspergillus niger*	4
*Aspergillus calidoustus*	10	*Aspergillus tamarii*	2	*Aspergillus parasiticus*	2
*Aspergillus carbonarius*	1	*Aspergillus unguis*	4	*Aspergillus terreus*	10
*Aspergillus carneus*	1	*Beauveria bassiana*	3	*Aspergillus versicolor*	3
*Aspergillus chevalieri*	4	*Cladosporium cladosporioides*	1	*Aureobasidium pullulans*	3
*Aspergillus creber*	1	*Curvularia lunata*	2	*Exophiala dermatitidis*	2
*Aspergillus dimorphicus*	1	*Epicoccum nigrum*	2	*Fusarium oxysporum*	21
*Aspergillus fischeri*	2	*Fusarium dimerum*	2	*Fusarium proliferatum*	10
*Aspergillus flavipes*	3	*Fusarium equiseti*	1	*Fusarium solani*	7
*Aspergillus floccosus*	3	*Fusarium verticillioides*	1	*Lichtheimia corymbifera*	2
*Aspergillus fumigatiaffinis*	2	*Galactomyces geotrichum*	2	*Microsporum canis*	2
*Aspergillus hiratsukae*	1	*Mucor circinelloides*	5	*Purpureocillium lilacinus*	3
*Aspergillus iizukae*	1	*Penicillium citrinum*	5	*Paecilomyces variotii*	3
*Aspergillus insuetus*	1	*Penicillium corylophilum*	4	*Penicillium chrysogenum*	27
*Aspergillus nomius*	1	*Penicillium funiculosum*	2	*Penicillium expansum*	2
*Aspergillus quadrilineatus*	1	*Penicillium purpurogenum*	1	*Penicillium glabrum*	9
*Aspergillus ruber*	2	*Penicillium rugulosum*	1	*Scedosporium apiospermum*	11
*Aspergillus subolivaceus*	1	*Rhizopus microsporus*	1	*Schizophyllum commune*	4
*Aspergillus tubingensis*	29	*Rhizopus oryzae*	6	*Scopulariopsis brevicaulis*	15
*Aspergillus westerdijkiae*	1	*Scedosporium aurantiacum*	6	*Trichophyton interdigitale*	1
*Cladosporium bruhnei*	1	*Scedosporium prolificans*	3	*Trichophyton rubrum*	8
*Cladosporium halotolerans*	2	*Trichoderma longibrachiatum*	2		
*Cladosporium pseudocladosporioides*	2				
*Cladosporium sphaerospermum*	1				
*Cochliobolus hawaiiensis*	2				
*Colletotrichum lineola*	1				
*Fomitopsis pinicola*	2				
*Fusarium sacchari*	1				
*Geosmithia argillacea*	1				
*Geosmithia pallida*	1				
*Hexagonia hydnoides*	1				
*Trichoderma orientale*	1				
*Lecythophora sp.*	1				
*Macrophomina phaseolina*	1				
*Microsporum audouinii*	1				
*Mucor hiemalis*	1				
*Penicillium asturianum*	1				
*Penicillium atramentosum*	1				
*Penicillium brasilianum*	3				
*Penicillium cecidicola*	2				
*Penicillium concentricum*	1				
*Penicillium crateriforme*	2				
*Penicillium crustosum*	3				
*Penicillium diversum*	1				
*Penicillium griseofulvum*	1				
*Penicillium helicum*	1				
*Talaromyces marnefei*	1				
*Penicillium nalgiovense*	1				
*Penicillium oxalicum*	1				
*Penicillium pinophilum*	3				
*Penicillium polonicum*	6				
*Penicillium raistrickii*	1				
*Penicillium rolfsii*	1				
*Penicillium sizovae*	2				
*Penicillium spinulosum*	2				
*Penicillium terrigenum*	1				
*Penicillium variabile*	2				
*Phaeosphaeria avenaria*	1				
*Phanerochaete chrysosporium*	2				
*Pithomyces chartarum*	3				
*Pleospora papaveracea*	1				
*Scedosporium boydii*	1				
*Pseudallescheria ellipsoidea*	1				
*Rhizomucor pusillus*	2				
*Rhizomucor variabilis*	2				
*Trichoderma reesei*	2				
*Tyromyces fissilis*	3				

### Identification of the strains from the assessment panel

#### MALDI-TOF MS-based identification

Liquid culture technology used in the Bruker protocol being not available in the two centers that participated to the study, culturing was performed on solid agar. After culturing the molds on solid Sabouraud Chloramphenicol Gentamicin agar plates (pH 5.6; glucose 40 g/L) (OXOID, Dardilly, France) for at least 48 h at 30 °C and the dermatophytes on solid Sabouraud Actidione (Bio Rad) at 27 °C, the strains were treated as described by Cassagne et al.[2], and as advised by the manufacturer : the colonies were gently scrapped using a scalpel blade, and the fungal material (approximately 2–3 mm in diameter) was suspended in a microtube containing 900 μL of anhydrous ethyl alcohol (Carlo Erba SDS, Val de Reuil, France) and 300 μL of sterile water (Water HPLC, Prolabo, BDH, Fontenay-sous-Bois, France). After a 10-min centrifugation step at 13,000 rpm, the pellet was resuspended in 10 μL of formic acid (Sigma-Aldrich, Lyon, France). After 5 min of incubation, 10 μL of acetonitrile (Prolabo BDH) was added. The suspension was then centrifuged at 13,000 rpm for 2 min, and four spots of 1 μL of protein extract per isolate were deposited. Last, the samples were covered with 1 μL of HCCA (α-cyano-4-hydroxycinnamic acid) matrix (Sigma-Aldrich, Lyon, France). MS acquisition was performed either in Marseille or Brussels using a Microflex LT system. All raw spectra were then centralized in Marseille and compared to the two reference databases. For all 1688 tested spectra (four spots for each strain) and each database, three possible scores were collected: the highest score corresponding to a true MS identification result at the species level if available, the highest score corresponding to a true identification at the genus level but false at the species level if available, and in all cases, the highest score false at the genus level.

#### DNA sequence-based identification

All strains belonging to the assessment panel were identified via DNA sequencing, which is the current gold standard in fungal identification. Nucleotide sequence analysis was performed either in Marseille (*n* = 224) or Brussels (*n* = 198). The rRNA ITS2 region was sequenced for each strain (using primer sequences ITS3 – GCA TCG ATG AAG AAC GCA GC and ITS4c – TCC TCC GCT TAT TGA TAT GC). An additional locus was analyzed to identify particular taxa: the partial beta-tubulin gene (primer sequences: Bt2A – GGT AAC CAA ATC GGT GCT GCT TTC and Bt2B – ACC CTC AGT GTA GTG ACC CTT GGC) was sequenced for *Aspergillus*, *Penicillium* and *Scedosporium* species, while the elongation factor (primer sequences: EF1 – ATG GGT AAG GAR GAC AAG AC and EF2 – GGA RGT ACC AGT SAT CAT GTT) was sequenced for *Fusarium* species. The hybridization temperatures applied for these primers were 54 °C for the ITS and beta-tubulin genes and 58 °C for the elongation factor gene. The DNA sequence-based identification criteria were as follows: a sequence longer than 350 base pairs and at least 99% homology with the NCBI and CBS nucleotide databases [35, 36].

### Identification algorithms

The following 10 distinct algorithms were used to identify the 422 isolates included in the panel.

A: Only one spot (i.e., the first of the four spots) is taken into account.
B: The first two spots are taken into account.
  B_1_: Only the identification corresponding to the higher of the two scores is taken into account, plotted and categorized.
  B_2_: To be accepted at the species level, both identifications corresponding to the two spots must be identical, and the same rule applies for the identification at the genus level.
C: The first three spots are taken into account.
  C_1_: Only the identification corresponding to the highest of the three scores is taken into account, plotted and categorized.
  C_2_: To be categorized as a concordant identification, the identification corresponding to the highest score must be identical to at least one of the other two identifications (at either the species or genus level).
  C_3_: To be categorized as a concordant identification, the three identifications corresponding to the three spots must be identical (at either the species or genus level).
D: All four spots are taken into account.
  D_1_: Only the identification corresponding to the highest of the four scores is taken into account, plotted and categorized.
  D_2_: To be categorized as a concordant identification, the identification corresponding to the highest score must be identical to at least one of the other three identifications (at either the species or genus level).
  D_3_: To be categorized as a concordant identification, the identification corresponding to the highest score must be identical to at least two of the other three identifications (at either the species or genus level).
  D_4_: To be categorized as a concordant identification, the four identifications corresponding to the four spots must be identical (at either the species or genus level).

Based on the individual spectrum results, 10 identification algorithms (or combinations of parameters) were tested per strain. For each spectrum, only the first identification (either true or false at the species and genus level) and the corresponding scores were collected. Each of the 10 algorithms was then tested using the identification thresholds 1.5, 1.6, 1.7, 1.8, 1.9, 2.0, 2.1, 2.2 and 2.3 for the entire panel consisting of 422 strains. The same process was applied for a reduced panel of 280 isolates that were represented at least three times in the in-house reference database and for another reduced panel of 191 isolates that were represented at least three times in the commercial database by Bruker.

### Statistical analyses

An initial identification assessment was carried out by categorizing the first identification for each spot into three classes: (1) concordant at the species level with the DNA-based identification, (2) concordant at the genus level but not at the species level with the DNA-based identification, or (3) discordant at the genus level with the DNA-based identification. We then determined the best rules for accepting identification at the genus and species levels. For each threshold, algorithm, panel and database, we calculated the percentage of submitted strains that fulfilled the criteria for identification as well as the positive predictive value (PPV) of the identification result at the genus and species levels.

## Results

### MALDI-TOF MS-based identification

**Fig. 1:**
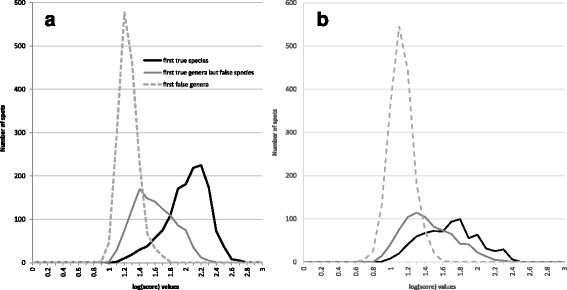
Highest log(score) value distribution of the 1688 tested spectra. **a** considering the first accurate species identification (*dark line*), the first accurate genus identification (*gray line*), and the first false genus identification (*dotted line*) using the in-house reference database. **b** considering the first accurate species identification (*dark line*), the first accurate genus identification (*gray line*), and the first false genus identification (*dotted line*) using the Bruker reference database

Using our in-house database to identify the 422 strains included in the panel, a clear separation was observed between the highest scores corresponding to true identifications at the species level and the highest scores corresponding to a false genus identification. Conversely, it was markedly more difficult to define a satisfying cut-off that differentiated between the highest scores corresponding to an accurate species identification and the highest scores corresponding to an accurate genus but false species identification (Fig. 1a). Using the commercial database led to a similar observation, although the separation between the three curves was less pronounced (Fig. 1b).

### Algorithm performance and identification score threshold

Analyses of the identification performances for the 422-strain panel with the Bruker database and the in-house database. The percentage of identified strains and PPVs at the species and genus levels obtained using the 10 distinct identification algorithms were assessed on the 422-strain panel using the Bruker database (Table 3) as well as the in-house reference database (Table 4).

**Table 3:** Comparison of the 10 different algorithms tested on the 422 strains included in the assessment panel against the Bruker reference database

Threshold		A	B1	B2	C1	C2	C3	D1	D2	D3	D4
LS 1.5	%accepted	43.71	50.36	38.95	47.74	47.03	31.83	51.31	51.31	44.66	30.40
	species PPV	0.46	0.47	0.43	0.51	0.51	0.46	0.50	0.50	0.48	0.42
	genus PPV	0.65	0.67	0.57	0.70	0.70	0.61	0.69	0.69	0.66	0.54
LS 1.6	%accepted	35.15	40.62	29.22	40.38	39.67	25.18	44.42	44.42	38.00	24.23
	species PPV	0.57	0.58	0.57	0.61	0.60	0.58	0.57	0.57	0.56	0.53
	genus PPV	0.80	0.83	0.76	0.83	0.83	0.77	0.79	0.79	0.78	0.68
LS 1.7	%accepted	28.27	33.73	22.33	33.49	32.78	19.48	**35.15**	35.15	29.45	16.39
	species PPV	0.71	0.70	0.74	0.73	0.72	0.76	**0.72**	0.72	0.73	0.78
	genus PPV	1.00	1.00	1.00	1.00	1.00	1.00	**1.00**	1.00	1.00	1.00
LS 1.8	%accepted	17.58	23.75	19.48	24.47	23.99	17.58	25.89	25.89	22.33	15.20
	species PPV	0.77	0.74	0.76	0.73	0.72	0.74	0.74	0.74	0.74	0.78
	genus PPV	1.00	1.00	1.00	1.00	1.00	1.00	1.00	1.00	1.00	1.00
LS 1.9	%accepted	11.88	16.63	14.25	16.39	15.91	12.59	17.58	17.58	15.68	12.11
	species PPV	0.78	0.77	0.77	0.77	0.76	0.75	0.77	0.77	0.74	0.78
	genus PPV	1.00	1.00	1.00	1.00	1.00	1.00	1.00	1.00	1.00	1.00
LS 2.0	%accepted	7.84	10.21	9.74	10.45	10.21	9.03	10.45	10.45	9.98	8.79
	species PPV	0.79	0.79	0.78	0.82	0.81	0.79	0.80	0.80	0.79	0.81
	genus PPV	1.00	1.00	1.00	1.00	1.00	1.00	1.00	1.00	1.00	1.00
LS 2.1	%accepted	4.51	5.46	4.99	5.94	5.94	5.46	5.70	5.70	5.70	5.23
	species PPV	0.79	0.78	0.76	0.80	0.80	0.78	0.75	0.75	0.75	0.77
	genus PPV	1.00	1.00	1.00	1.00	1.00	1.00	1.00	1.00	1.00	1.00
LS 2.2	%accepted	3.33	4.04	3.56	3.56	3.56	3.09	3.80	3.80	3.80	3.56
	species PPV	0.86	0.88	0.87	0.87	0.87	0.85	0.81	0.81	0.81	0.80
	genus PPV	1.00	1.00	1.00	1.00	1.00	1.00	1.00	1.00	1.00	1.00
LS 2.3	%accepted	2.14	2.38	1.90	1.19	1.19	0.95	1.19	1.19	1.19	0.95
	species PPV	0.78	0.80	0.75	1.00	1.00	1.00	1.00	1.00	1.00	1.00
	genus PPV	1.00	1.00	1.00	1.00	1.00	1.00	1.00	1.00	1.00	1.00

% accepted: percentage of submitted strains that fulfilled the criteria for identification at either the genus or species level, depending on the applied threshold; species PPV: positive predictive value at the species level; genus PPV: positive predictive value at the genus level

**Table 4:** Comparison of the 10 different algorithms tested on the 422 strains included in the assessment panel against the in-house reference database

Threshold		A	B1	B2	C1	C2	C3	D1	D2	D3	D4
LS 1.5	%accepted	90.97	93.35	81.00	92.64	91.69	73.63	94.54	94.30	88.36	72.45
	species PPV	0.80	0.81	0.82	0.83	0.83	0.85	0.82	0.82	0.82	0.83
	genus PPV	0.90	0.92	0.90	0.93	0.93	0.93	0.92	0.92	0.92	0.91
LS 1.6	%accepted	87.41	90.02	77.67	89.55	88.60	70.78	90.26	90.02	84.32	68.65
	species PPV	0.84	0.84	0.86	0.85	0.86	0.89	0.86	0.86	0.86	0.88
	genus PPV	0.94	0.95	0.94	0.96	0.96	0.96	0.97	0.97	0.97	0.96
LS 1.7	%accepted	81.95	85.27	73.16	85.99	85.27	68.17	**87.41**	87.17	81.47	65.80
	species PPV	0.89	0.89	0.91	0.89	0.89	0.92	**0.89**	0.89	0.89	0.92
	genus PPV	1.00	1.00	1.00	1.00	1.00	1.00	**1.00**	1.00	1.00	1.00
LS 1.8	%accepted	76.96	81.00	71.97	79.33	78.86	65.56	81.71	81.47	77.20	64.61
	species PPV	0.89	0.89	0.91	0.90	0.91	0.92	0.90	0.90	0.90	0.92
	genus PPV	1.00	1.00	1.00	1.00	1.00	1.00	1.00	1.00	1.00	1.00
LS 1.9	%accepted	68.88	74.11	67.46	70.78	70.31	61.28	74.11	73.87	70.55	60.57
	species PPV	0.90	0.89	0.90	0.90	0.91	0.92	0.89	0.89	0.89	0.91
	genus PPV	1.00	1.00	1.00	1.00	1.00	1.00	1.00	1.00	1.00	1.00
LS 2.0	%accepted	56.77	63.18	57.72	63.18	62.71	55.58	63.66	63.42	61.28	55.11
	species PPV	0.92	0.91	0.92	0.91	0.92	0.92	0.91	0.91	0.91	0.91
	genus PPV	1.00	1.00	1.00	1.00	1.00	1.00	1.00	1.00	1.00	1.00
LS 2.1	%accepted	43.47	50.36	46.08	49.64	49.41	43.94	50.59	50.36	48.93	44.18
	species PPV	0.94	0.92	0.93	0.92	0.93	0.93	0.92	0.92	0.92	0.92
	genus PPV	1.00	1.00	1.00	1.00	1.00	1.00	1.00	1.00	1.00	1.00
LS 2.2	%accepted	29.69	35.15	32.30	33.02	32.78	29.93	34.44	34.20	33.97	31.12
	species PPV	0.95	0.93	0.93	0.92	0.93	0.94	0.92	0.92	0.92	0.92
	genus PPV	1.00	1.00	1.00	1.00	1.00	1.00	1.00	1.00	1.00	1.00
LS 2.3	%accepted	14.25	18.29	17.34	16.86	16.86	16.15	18.05	18.05	18.05	17.34
	species PPV	0.95	0.95	0.95	0.93	0.93	0.93	0.92	0.92	0.92	0.92
	genus PPV	1.00	1.00	1.00	1.00	1.00	1.00	1.00	1.00	1.00	1.00

% accepted: percentage of submitted strains that fulfilled the criteria for identification at either the genus or species level, depending on the applied threshold; species PPV: positive predictive value at the species level; genus PPV: positive predictive value at the genus level

Notable differences in successful identification rates were highlighted between the 10 algorithms (up to 22% difference between the best identification rate and the worst one for one defined threshold). In particular, with both references databases, the algorithm that selected the best results among four spots (D1) increased the accepted identification rate (i.e., percentage of assessed strains that fulfilled the criteria for identification at either the genus or species level) (A < B1 < C1 < D1) without notably changing the identification PPV. By contrast, the inter-spot concordance criteria did not increase the identification PPV at the species level and reduced the accepted result rate (D4 < D3 < D2 ≈ D1). The main difference between the results obtained with the two databases was the percentage of accepted identifications, which was markedly lower with the Bruker database than with the in-house reference database (up to 59.8% difference between the two databases). Additionally, while the PPV for genus level identification was 100% for both databases using the 1.7 threshold, regardless of the other decision criteria applied, the PPV for species level identification was better with our in-house database than with the Bruker database (from 0.13 units for the D4 algorithm to 0.18 units for the A1 and B1 algorithms). Using the in-house reference database, the D1 combination with a threshold at 1.7 yielded 87.41% of accepted identifications, with a species identification PPV of 0.89.

We noted an increase in the species PPV with threshold values higher than 1.7, but the loss in percentage of identification is important. Using the identification score threshold of 2.00, as recommended by the manufacturer, led to a 23.75% decrease in accepted identifications and only a 2% increase in the PPV. The “highly probable species identification” criterion by Bruker (i.e., a score above the threshold of 2.3) yielded a species PPV of 0.92 but only 18.05% of accepted identifications (76 strains) using our in-house database. The identification results were even less acceptable when using the commercial database, with only 44 strains (10.45%) reaching the 2.0 threshold and 5 strains (1.19%) reaching the 2.3 threshold.

#### Description of the misidentifications obtained with both databases

For 32 strains, the identification best score using the D1 algorithm was higher than the 1.7 threshold, although the species level result was incorrect using the in-house database. For all of these strains, proper identification at the species level was impeded by the close similarity of the MALDI-TOF MS spectra of some species belonging to the same species complex (as it is the case with *Fusarium solani* and its closely related species *Fusarium petrolophilum*). The details of these strains are presented in Table 5. The same finding was observed for 39 misidentifications obtained using the Bruker database (Table 6).

**Table 5:** List of the 39 identification failures obtained using the Bruker database and the D1 algorithm with a score above the 1.7 threshold

Identification via molecular biology	Nb of occurrences	Identification D1	Score D1	Number of references in the Bruker reference database
*Aspergillus tubingensis*	10	*Aspergillus niger*	2.329/2.161/2.102/1.928/2.238/1.955/2.333/1.983/1.987/1.829	0
*Fusarium proliferatum*	2	*Fusarium verticillioides*	1.845/1.718	5
*Curvularia hawaiensis*	2	*Curvularia pallescens*	1.879/2.079	0
*Penicillium brasilianum*	2	*Penicillium discolor*	1.92/1.887	0
*Arthrinium arundinis*	1	*Arthrinium phaeospermum*	1.883	0
*Aspergillus quadrilineatus*	1	*Aspergillus nidulans*	2.065	0
*Aspergillus calidoustus*	4	*Aspergillus ustus*	1.775/2.076/1.771/1.731	0
*Fusarium sacchari*	1	*Fusarium proliferatum*	1.742	0
*Aspergillus subolivaceus*	1	*Aspergillus flavus*	1.985	0
*Scopulariopsis brevicaulis*	2	*Scopulariopsis brumptii*	1.781/1.925	9
*Fusarium verticillioides*	1	*Fusarium proliferatum*	1.953	2
*Curvularia lunata*	1	*Curvularia pallescens*	2.039	1
*Aspergillus creber*	1	*Aspergillus versicolor*	2.103	0
*Aspergillus sydowii*	2	*Aspergillus versicolor*	1.884/1.897	1
*Aspergillus chevalieri*	2	*Aspergillus hollandicus*	1.84/1.766	0
*Fusarium equiseti*	1	*Fusarium incarnatum*	1.701	1
*Trichophyton rubrum*	1	*Trichophyton mentagrophytes*	1.701	6
*Aspergillus alabamensis*	1	*Aspergillus terreus*	1.739	0
*Aspergillus fischeri*	1	*Aspergillus fumigatus*	1.898	0
*Trichoderma orientale*	1	*Trichoderma longibrachiatum*	1.802	0
*Trichoderma reesei*	1	*Trichoderma koningii*	1.865	0

**Table 6:** List of the 32 identification failures obtained using the in-house database and the D1 algorithm with a score above the 1.7 threshold

Identification via molecular biology	Nb of occurrences	Identification D1	Score D1	Number of references in the in-house reference database
*Acremonium strictum*	3	*Acremonium sclerotigenum*	1.952/2.042/2.126	1
*Alternaria alternata*	1	*Alternaria soliaegyptiaca*	2.211	1
*Aspergillus sclerotiorum*	1	*Aspergillus persii*	1.828	2
*Cladosporium halotolerans*	1	*Cladosporium sphaerospermum*	1.975	0
*Cladosporium pseudocladosporioides*	1	*Cladosporium cladosporioides*	1.969	1
*Aspergillus nidulans*	1	*Aspergillus echinulatus*	2.203	5
*Aspergillus quadrilineatus*	1	*Aspergillus nidulans*	2.339	2
*Aspergillus hollandicus*	1	*Aspergillus heterocaryoticus*	2.496	1
*Aspergillus rubrobrunneus*	1	*Aspergillus hollandicus*	2.325	1
*Fusarium solani*	2	*Fusarium petroliphilum*	1.981/2.081	7
*Fusarium solani*	1	*Acremonium falciforme*	2.036	7
*Trichoderma orientale*	1	*Trichoderma longibrachiatum*	2.026	0
*Mucor irregularis*	1	*Rhizomucor variabilis*	2.235	0
*Penicillium asturianum*	1	*Penicillium oxalicum*	1.953	1
*Penicillium cecidicola*	1	*Penicillium funiculosum*	2.067	1
*Penicillium chrysogenum*	1	*Penicillium nalgiovense*	2.328	6
*Penicillium glabrum*	1	*Penicillium purpurescens*	2.249	4
*Penicillium nalgiovense*	1	*Penicillium chrysogenum*	2.17	3
*Talaromyces purpurogenus*	1	*Talaromyces amestolkiae*	2.39	1
*Penicillium rubrum*	1	*Talaromyces amestolkiae*	2.069	2
*Pleospora papaveracea*	1	*Ulocladium oudemansii*	2.115	0
*Pseudallescheria boydii*	1	*Scedosporium apiospermum*	1.721	3
*Scedosporium apiospermum*	2	*Scedosporium boydii*	1.746/2.249	5
*Scopulariopsis acremonium*	1	*Scopulariopsis brevicaulis*	2.178	1
*Talaromyces ramulosus*	1	*Penicillium cecidicola*	2.375	0
*Talaromyces stollii*	1	*Penicillium funiculosum*	2.13	2
*Trichoderma reesei*	1	*Trichoderma citrinoviride*	1.727	0
*Trichophyton rubrum*	1	*Trichophyton violaceum*	1.95	12

#### Analyses of the identification performances for the restricted panels with the Bruker database and the in-house database

The robustness of the identification algorithms was tested by performing the same analysis on a restricted panel including only the strains that were represented by at least three distinct strains in the reference spectrum libraries (the restricted panel used with the Bruker database (*n* = 191) is different than the one used with the in-house database (*n* = 280) due to the differences in databases composition). As expected, the results were better than those obtained with the 422-strain panel (Tables 7 and 8), with a higher percentage of results reaching the thresholds (from 3.30% increase in percentage of accepted identification with B1 to 24.94% increase with D4 when selecting the 1.7 threshold) and a reduced number of misidentifications. For example, using the in-house database, the species PPV for the D1 algorithm with a 1.7 score threshold on the comprehensive 422-strain panel increased from 0.89 to 0.97 when testing a sub-panel including only the 280 strains for which species were represented by at least three distinct strains. Similar trends were observed when using the commercial database (increase in the species PPV from 0.72 to 0.93).

**Table 7:** Performance comparison of the 10 different algorithms tested on the sub-panel of 191 strains that are represented by three or more strains in the Bruker reference library

Threshold		A1	B1	B2	C1	C2	C3	D1	D2	D3	D4
LS 1.5	%accepted	56.02	61.26	48.17	58.12	57.07	38.74	63.35	63.35	53.93	35.08%
	species PPV	0.63	0.66	0.59	0.71	0.71	0.64	0.69	0.69	0.66	0.58
	genus PPV	0.69	0.72	0.64	0.75	0.74	0.68	0.74	0.74	0.72	0.60
LS 1.6	%accepted	46.07	49.74	36.65	49.74	48.69	31.41	56.54	56.54	47.64	28.80
	species PPV	0.76	0.81	0.77	0.83	0.83	0.78	0.77	0.77	0.75	0.71
	genus PPV	0.84	0.88	0.84	0.87	0.87	0.83	0.82	0.82	0.81	0.73
LS 1.7	%accepted	38.74	43.98	30.89	43.46	42.41	26.18	**46.60**	46.60	38.74	20.94
	species PPV	0.91	0.92	0.92	0.95	0.95	0.94	**0.93**	0.93	0.92	0.98
	genus PPV	1.00	1.00	1.00	1.00	1.00	1.00	**1.00**	1.00	1.00	1.00
LS 1.8	%accepted	24.61	31.94	27.23	31.94	31.41	23.04	33.51	33.51	28.80	18.32
	species PPV	0.98	0.95	0.94	0.97	0.97	0.95	0.98	0.98	0.98	1.00
	genus PPV	1.00	1.00	1.00	1.00	1.00	1.00	1.00	1.00	1.00	1.00
LS 1.9	%accepted	17.28	23.04	19.90	21.47	20.94	15.71	23.56	23.56	20.42	15.71
	species PPV	0.97	0.95	0.95	1.00	1.00	1.00	1.00	1.00	1.00	1.00
	genus PPV	1.00	1.00	1.00	1.00	1.00	1.00	1.00	1.00	1.00	1.00
LS 2.0	%accepted	12.04	15.71	14.66	14.14	14.14	11.52	13.61	13.61	13.09	11.52
	species PPV	1.00	0.97	0.96	1.00	1.00	1.00	1.00	1.00	1.00	1.00
	genus PPV	1.00	1.00	1.00	1.00	1.00	1.00	1.00	1.00	1.00	1.00
LS 2.1	%accepted	6.81	7.85	6.81	8.38	8.38	7.33	7.33	7.33	7.33	6.81
	species PPV	1.00	1.00	1.00	1.00	1.00	1.00	1.00	1.00	1.00	1.00
	genus PPV	1.00	1.00	1.00	1.00	1.00	1.00	1.00	1.00	1.00	1.00
LS 2.2	%accepted	5.24	6.81	5.76	5.76	5.76	4.71	5.24	5.24	5.24	4.71
	species PPV	1.00	1.00	1.00	1.00	1.00	1.00	1.00	1.00	1.00	1.00
	genus PPV	1.00	1.00	1.00	1.00	1.00	1.00	1.00	1.00	1.00	1.00
LS 2.3	%accepted	3.14	3.66	2.62	2.09	2.09	1.57	2.09	2.09	2.09	1.57
	species PPV	1.00	1.00	1.00	1.00	1.00	1.00	1.00	1.00	1.00	1.00
	genus PPV	1.00	1.00	1.00	1.00	1.00	1.00	1.00	1.00	1.00	1.00

% accepted: percentage of submitted strains that fulfilled the criteria for identification at either the genus or species level depending on the applied threshold; species PPV: positive predictive value at the species level; genus PPV: positive predictive value at the genus level

**Table 8:** Comparison of the 10 different algorithms tested on a sub-panel of 280 strains that are represented by three or more strains in the in-house reference library

Threshold		A	B1	B2	C1	C2	C3	D1	D2	D3	D4
LS 1.5	%accepted	93.57	96.07	95.36	97.14	97.14	95.36	98.21	98.21	97.14	95.71
	species PPV	0.93	0.94	0.92	0.94	0.94	0.91	0.95	0.93	0.92	0.90
	genus PPV	1.00	1.00	1.00	1.00	1.00	1.00	1.00	1.00	1.00	1.00
LS 1.6	%accepted	90.00	92.50	92.50	93.93	93.93	93.21	95.36	95.36	94.64	93.93
	species PPV	0.94	0.94	0.92	0.95	0.95	0.91	0.96	0.95	0.93	0.91
	genus PPV	1.00	1.00	1.00	1.00	1.00	1.00	1.00	1.00	1.00	1.00
LS 1.7	%accepted	86.07	88.57	88.57	91.07	91.07	90.36	**92.14**	92.14	91.43	90.71
	species PPV	0.95	0.95	0.92	0.96	0.95	0.92	**0.97**	0.95	0.94	0.91
	genus PPV	1.00	1.00	1.00	1.00	1.00	1.00	**1.00**	1.00	1.00	1.00
LS 1.8	%accepted	81.07	82.86	82.86	83.93	83.93	83.21	86.07	86.07	85.71	85.36
	species PPV	0.96	0.96	0.93	0.97	0.97	0.93	0.97	0.96	0.95	0.92
	genus PPV	1.00	1.00	1.00	1.00	1.00	1.00	1.00	1.00	1.00	1.00
LS 1.9	%accepted	69.64	75.36	75.36	76.79	76.79	76.07	79.29	79.29	78.93	78.57
	species PPV	0.97	0.96	0.95	0.97	0.97	0.93	0.97	0.96	0.95	0.93
	genus PPV	1.00	0.99	1.00	1.00	1.00	1.00	1.00	1.00	1.00	1.00
LS 2.0	%accepted	60.00	65.00	65.00	68.57	68.57	67.86	70.36	70.36	70.00	69.64
	species PPV	0.98	0.97	0.95	0.97	0.97	0.94	0.97	0.96	0.95	0.94
	genus PPV	1.00	0.99	1.00	1.00	1.00	1.00	1.00	1.00	1.00	1.00
LS 2.1	%accepted	46.79	54.64	54.64	57.14	57.14	56.79	59.64	59.64	59.29	59.29
	species PPV	0.98	0.97	0.95	0.98	0.98	0.94	0.98	0.97	0.96	0.94
	genus PPV	1.00	1.00	1.00	1.00	1.00	1.00	1.00	1.00	1.00	1.00
LS 2.2	%accepted	30.36	36.79	36.79	40.00	40.00	39.64	42.50	42.50	42.14	42.14
	species PPV	0.99	0.97	0.95	0.97	0.97	0.95	0.98	0.97	0.97	0.96
	genus PPV	1.00	1.00	1.00	1.00	1.00	1.00	1.00	1.00	1.00	1.00
LS 2.3	%accepted	15.71	19.29	19.29	21.43	21.43	21.43	22.50	22.50	22.50	22.50
	species PPV	0.97	0.98	0.96	0.97	0.97	0.95	0.97	0.95	0.95	0.95
	genus PPV	1.00	1.00	1.00	1.00	1.00	1.00	1.00	1.00	1.00	1.00

% accepted: percentage of submitted strains that fulfilled the criteria for identification at either the genus or species level depending on the applied threshold; species PPV: positive predictive value at the species level; genus PPV: positive predictive value at the genus level

Twenty-two species (180 isolates) of this study are represented by at least three references in both databases. We performed a per species comparison of the efficiency of the two databases for those 22 species, using the D1 algorithm. Results are compiled in Table 9. For the total of the 180 isolates, the percentage of accepted identifications is lower with the Bruker database than with the in-house database (47.22% vs 91.67%). The comparison of the species PPV obtained with both databases highlighted weaknesses for four species (*A. alternata, F. solani, P. chrysogenum* and *S. apiospermum*) with the in-house database (due to misidentifications with closely related species), and for five species (*A. parasiticus, F. proliferatum, S. brevicaulis, T. interdigitale* and *T. rubrum*) with the Bruker database. The species PPV was equal to 1.00 and identical between the two databases for 13 species of this group.

**Table 9:** Comparison of the identification efficiency of the two databases per species, using the D1 algorithm of identification with a 1.7 LS threshold, for 180 strains that are represented by three or more strains in both reference databases

		In-House database		Bruker database	
	nb of isolates	% accepted	species PPV	% accepted	species PPV
*Absidia corymbifera*	2	100.00	1.00	50.00	1.00
*Alternaria alternata*	2	100.00	0.50	100.00	1.00
*Aspergillus flavus*	15	100.00	1.00	73.33	1.00
*Aspergillus fumigatus*	20	95.00	1.00	45.00	1.00
*Aspergillus nidulans*	8	100.00	1.00	75.00	1.00
*Aspergillus niger*	4	100.00	1.00	50.00	1.00
*Aspergillus parasiticus*	2	100.00	1.00	0.00	Not Relevant
*Aspergillus terreus*	10	100.00	1.00	30.00	1.00
*Aspergillus versicolor*	3	66.67	1.00	33.33	1.00
*Exophiala dermatitidis*	2	50.00	1.00	50.00	1.00
*Fusarium oxysporum*	21	80.95	1.00	19.05	1.00
*Fusarium proliferatum*	10	100.00	1.00	60.00	0.33
*Fusarium solani*	7	57.14	0.25	28.57	1.00
*Microsporum canis*	2	100.00	1.00	50.00	1.00
*Purpureocillium lilacinus*	3	66.67	1.00	66.67	1.00
*Paecilomyces variotii*	3	66.67	1.00	66.67	1.00
*Penicillium chrysogenum*	27	100.00	0.96	33.33	1.00
*Scedosporium apiospermum*	11	100.00	0.82	81.82	1.00
*Schizophyllum commune*	4	100.00	1.00	100.00	1.00
*Scopulariopsis brevicaulis*	15	86.67	1.00	46.67	0.71
*Trichophyton interdigitale*	1	100.00	1.00	0.00	Not Relevant
*Trichophyton rubrum*	8	87.50	0.86	37.50	0.67
Total Isolates	180	91.67	0.95	47.22	0.92

Nb of isolates : number of isolates from the panel of strain that belong to each species. % accepted: percentage of submitted strains that fulfilled the criteria for identification at either the genus or species level; species PPV: positive predictive value at the species level

## Discussion

As shown in this study and several others, MALDI-TOF MS has recently been optimized to identify filamentous fungi at the species level, provided that an appropriate database is available [2, 4, 6–8]. The assessment panel used in this study comprised isolates belonging to 126 different species, including many difficult-to-identify isolates. Consequently, the identification rate was low compared with previously published studies, especially studies utilizing the commercial database. Using such a challenging panel, it was possible to define a threshold discriminating correct identifications at the species level from false identifications at the genus level, although identifying a threshold that differentiated between a correct and false identification at the species level among closely related species was not feasible. Our findings indicate that the best identification results (PPV at the species level and percentage of accepted identifications) were obtained when applying a decisional algorithm in which only the highest score of four spots was taken into account, and the identification was accepted if the score was at least 1.7. This threshold is notably lower than that recommended for bacteria identification, and has already been advised by several authors for yeast identification [32, 33]. Furthermore, we also observed a marked decrease in percentage of identification between a threshold of 1.7 and 2.0. When the threshold was set too high, we noted a 20% loss in identification efficiency and only a small benefit of 2% in the differentiation of certain close species. Even if the Bruker commercial database yielded a markedly lower percentage of accepted identifications (probably due to the fact that the database was built using liquid culture media, that is does not frequently involve more than two or three references per species, and that there are errors of reference labeling), the PPV remained correct for the spectra reaching the 1.7 threshold. This result corroborates the findings of several authors who have suggested that lowering the identification score thresholds would improve MALDI-TOF MS-based fungal identification efficiency [7, 17, 32, 33]. However, we strongly advise against a threshold below 1.7, which could yield an increase in false identifications, in particular at the genus level, as shown in Tables 3 and 4.

The major problem encountered with the data set obtained from the assessment panel was the resemblance between the MALDI-TOF MS spectra of some species. For 32 strains, the similarity in MALDI-TOF MS spectra yielded an incorrect identification at the species level, although these identification errors systematically corresponded to taxonomically close species often belonging to the same species complex (except for *Pleospora papaveracea*, which was identified as *Ulocladium oudemansi*). Among these 32 strains (27 species), only a few species are known to be pathogenic to humans, of which none are major human pathogens. Regarding the pathogenic species that were misidentified in our study, the treatment would not vary from that prescribed based on molecular identification, and the differentiation of these species is not clinically relevant. The remaining species are often considered to be a result of contamination, and their antifungal susceptibility is unknown. Interestingly, raising the identification score threshold to 2.3 did not entirely resolve this identification issue of taxonomically close species, as six strains were incorrectly characterized despite a score above 2.3, even using our extensive in-house database. Thus, in our study, one could question the relevance of capacity and necessity to distinguish between these closely related species as none of the misidentifications implied a change in treatment.

Our results also show that the key step in efficient identification was to increase the number of replicate spots per strain. Testing four spots of each filamentous fungus yielded better identification performance, with an increase of 5% to 7% of accepted strain identifications. Such spot replications did not markedly increase the workload, as it took only a few seconds to perform the additional spots. However, the replicates may increase the per-strain acquisition time in the laboratory workflow. The four-spot method has been applied in the routine workflow at the Marseille Mycology Laboratory for the past five years, which demonstrates that this procedure is easily applicable in hospital laboratories to identify filamentous fungi isolated from clinical samples and from hospital environment samples. Multiplying the number of deposits per strains imply a small increase in the cost per strain as only one reagent (HCCA matrix) has to be used more extensively. The decisional algorithm implying concordance of the identification results of two to four spots did not enhance identification performance, regardless of the threshold selected. Furthermore, the D2, D3 and D4 algorithms tended to lower the percentage of validated identifications by disregarding identification results that would have been correct using the D1 decisional algorithm.

When focusing on the species that were represented by at least three distinct strains in the reference spectrum library, identification efficacy markedly increased. This result stresses the importance of a comprehensive reference spectrum library for the identification of filamentous fungi, including dermatophytes, as previously reported by our group [6], and as previously demonstrated for yeasts [32]. The multiplication of strains per species as well as subcultures per strain allowed us to cover the variations seen within the environment for filamentous fungi.

Our in-house reference database, which yielded markedly better results compared with the commercial Bruker database, was constructed mainly with collection strains and with some clinical strains isolated in our laboratory and obtained during routine analyses. These clinical strains were later included in the BCCM/IHEM collection in Brussels. Nevertheless, even our enhanced database still requires optimization to identify rare species. Indeed, the poor representation of rare species in the database may explain the identification percentage of 87.4% obtained with this challenging panel of 422 strains (92.1% with the 280 strains represented by more than three strains in the reference database), compared with the extremely good identification results of common species (98.1%) exposed by Gautier et al. [21].

## Conclusion

The growing number of publications on MALDI-TOF MS-based fungal identification indicates that this technology is widely used and applicable to the routine laboratory workflow. MALDI-TOF MS-based identification of fungi yields exceedingly more accurate results compared with morphology-based analyses [21]. Furthermore, this technology is less expensive and easier than the current DNA based-identification gold standard, with a turnaround time allowing achieving the analysis more rapidly. Currently, the major limitation of this approach is the lack of a comprehensive and efficient reference spectrum library. We acknowledge that in-house reference databases that are non-open-source, such as ours, are of little benefit to the global scientific community. Therefore, we plan to deploy an accessible version of our database along with an identification algorithm that can be queried online to assist scientific teams to expand the identification of fungal species. Experiments implying a multicenter approach are ongoing to finalize this project.

## Additional file

Additional file 1: Table S1. The species included in the in-house reference database (as labeled by the BCCM/IHEM or Marseille Mycology Laboratory staff at the time of database development). Nb str: number of strains per genus; nb sp: number of species belonging to each genus. The number in brackets represents the number of strains corresponding to each species. (DOCX 52 kb)
