# Supplementary material for: Decision criteria for MALDI-TOF MS-based identification of filamentous fungi using commercial and in-house reference databases
Source: BMC Microbiol. 2017 Jan 31;17:25. doi: 10.1186/s12866-017-0937-2 (PMC5282874; doi:10.1186/s12866-017-0937-2)
Supplement: Additional file 1: Table S1. — The species included in the in-house reference database (as labeled by the BCCM/IHEM or Marseille Mycology Laboratory staff at the time of database development). Nb str: number of strains per genus; nb sp: number of species belonging to each genus. The number in brackets represents the number of strains corresponding to each species. (DOCX 52 kb) [file 12866_2017_937_MOESM1_ESM.docx]

Supplementary Table 1: The species included in the in-house reference database (as labeled by the BCCM/IHEM or Marseille Mycology Laboratory staff at the time of database development). Nb str: number of strains per genus; nb sp: number of species belonging to each genus. The number in brackets represents the number of strains corresponding to each species.

| Genera | nb str | nb sp | Detail of species included |
| --- | --- | --- | --- |
| *Absidia* | 10 | 5 | *Absidia corymbifera (6) -Absidia cylindrospora (1) -Absidia glauca (1) -Absidia pseudocylindrospora (1) -Absidia spinosa (1)* |
| *Acrodontium* | 5 | 3 | *Acrodontium crateriforme (2) -Acrodontium salmoneum (2) -Acrodontium simplex (1)* |
| *Actinomucor* | 2 | 2 | *Actinomucor elegans (1) -Actinomucor elegans var meitauzae (1)* |
| *Alternaria* | 17 | 10 | *A.acalyphicola (1) -A.alternata (7) -A.calycipiricola (1) -A.chlamydospora (1) -A.citri (1) -A.infectoria (2) -A.malorum (1) -A.roseogrisea (1) -A.soliaegyptiaca (1) -A.undulata (1)* |
| *Amaurascopsis* | 2 | 2 | *Amaurascopsis perforata (1) -Amaurascopsis perforatus (1)* |
| *Amauroascus* | 2 | 1 | *Amauroascus kuehnii (2)* |
| *Anixiopsis* | 1 | 1 | *Anixiopsis biplanata (1)* |
| *Anthopsis* | 1 | 1 | *Anthopsis deltoidea (1)* |
| *Aphanoascus* | 3 | 2 | *Aphanoascus fulvescens (2) -Aphanoascus verrucosum (1)* |
| *Aphanocladium* | 1 | 1 | *Aphanocladium album (1)* |
| *Apophysomyces* | 1 | 1 | *Apophysomyces variabilis (1)* |
| *Arachniotus* | 1 | 1 | *Arachniotus littoralis (1)* |
| *Arthrinium* | 5 | 4 | *Arthrinium arundinis (2) -Arthrinium marii (1) – Arthrinium sphaerospermum (1) – Arthrinium rasikravindrii (1)* |
| *Arthrobotrys* | 1 | 1 | *Arthrobotrys oligospora (1)* |
| *Arthrographis* | 1 | 1 | *Arthrographis kalrae (1)* |
| *Ascochyta* | 1 | 1 | *Ascochyta pisi var pisi (1)* |
| *Aspergillus /  Emericella /  Eurotium /*  *Neosartorya* | 328 | 120 | *A.aculeatus (3) –A. af.viridinutans (1) - A.alabamensis (3) -A.allahabadi (2) -A.alliaceus (4) -A.amylovorus (1) – A.ardalensis (1) – A.astellatus (1) – A.auricomus (1) -A.aureolatus (1) – A.aureoterreus (1) - A.austroafricanus (1) -A.avenaceus (1) -A.bombycis (1) -A.brasiliensis (2) -A.brevipes (1) -A.caelatus (2) -A.caesiellus (2) -A.caespitosus (1) -A.calidoustus (6) -A.candidus (6) -A.carbonarius (3) -A.carneus (3) -A.cervinus (1) -A.clavatoflavus (1) -A.clavatus (4) -A.coremiiformis (1) -A.creber (4) -A.cretensis (1) -A.dimorphicus (1) -A.duricaulis (1) -A.eburneocremeus (1) -A.ellipticus (1) -A.flaschentraegeri (1) -A.flavipes (2) -A.flavipes ssp (1) -A.flavofurcatus (1) -A.flavus (27) -A.flavus var columnaris (1) -A.floccosus (1) -A.fumigatiaffinis (1) -A.fumigatus (14) -A.fumigatus var ellipticus (2) -A.giganteus (1) -A.heterocaryoticus (1) -A.heteromorphus (2) -A.hollandicus (8) – A. hortai (1) - A.iiukae (1) -A.insuetus (4) -A.insulicola (1) -A.japonicus (5) -A.jensenii (1) -A.lentulus (3) -A.melleus (3) -A.microcysticus (1) -A.nidulans (5) -A.niger (30) -A.niveus (1) -A.nomius (6) -A.ochraceus (5) -A.oryzae (4) -A.ostianus (1) -A.parasiticus (3) -A.persii (4) -A.proliferans (1)- A.protuberus (1) – A.pseudoglaucus (1) -A.pseudoustus (2) -A.puniceus (2) -A.puulaauensis (2) -A.restrictus (2) -A.sclerotiorum (2) -A.sojae (2) -A.sparsus (1) -A.spelunceus (2) -A.stromatoides (1) -A.subolivaceus (1) -A.sydowii (4) -A.tabacinus (2) -A.tamarii (6) -A.terreus (12) -A.terreus var africanus (1) -A.thomii (1) -A.tubingensis (17) -A.unguis (2) -A.ustus var pseudodeflectus (1) -A.uvarum (1) -A.versicolor (4) -A.viridinutans (1) -A.wentii (1) -A.westerdijkiae (3) - E.desertorum (1) -E.echinulata (4) -E.heterothallica (2) -E.nidulans var acristata (1) -E.quadrilineata (2) -E.rugulosa (1) -E.striata (1) -E.athecium (1) -E.chevalieri (3) -E.cristatum (2) -E.echinulatum (1) -E.herbariorum (1) -E.intermedius (1) -E.minus (1) -E.montevidense (1) -E.pseudoglaucum (1) -E.repens (1) -E.rubrum (1) -N.aurata (1) -N.aureola (3) -N.fischeri (2) -N.glabra (1) -N.hiratsukae (4) -N.pseudofischeri (3) -N.quadricincta (1) -N.stramenia (1) – N.udagawae (3)* |
| *Aureobasidium* | 4 | 2 | *Aureobasidium pullulans (2) -Aureobasidium pullulans var melanogenum (2)* |
| *Auxarthron* | 5 | 5 | *Auxarthron alboluteum (1) – Auxarthron californiense (1) -Auxarthron conjugatum (1) -Auxarthron reticulatum (1) -Auxarthron umbrinum (1)* |
| *Beauveria* | 10 | 2 | *Beauveria bassiana (9) -Beauveria caledonica (1)* |
| *Bjerkandera* | 1 | 1 | *Bjerkandera adusta (1)* |
| *Blastobotrys* | 1 | 1 | *Blastobotrys proliferans (1)* |
| *Botrytis* | 1 | 1 | *Botrytis anthophila (1)* |
| *Chaetomium* | 2 | 1 | *Chaetomium globosum (2)* |
| *Chalaropsis* | 1 | 1 | *Chalaropsis punctulata (1)* |
| *Chrysonilia* | 2 | 1 | *Chrysonilia tetrasperma (2)* |
| *Chrysosporium* | 9 | 7 | *C. keratinophilum (2) –C. lucknowense (1) –C. merdarium (1) –C. pannicola (1) –C. queenslandicum (1) –C. synchronum (1) –C. tropicum (2)* |
| *Cladiophiolophora* | 1 | 1 | *Cladiophiolophora carrionii (1)* |
| *Cladobotryum* | 1 | 1 | *Cladobotryum mycophilum (1)* |
| *Cladosporium* | 12 | 9 | *C. cladosporioides (2) - C.cucumerinum (1) -C.macrocarpum (1) -C.oxysporum (1) -C.pseudocladosporioides (1) -C.ramotenellum (1) - C.sphaerospermum (3) -C.tenuissimum (1) -C.variabile (1)* |
| *Claviceps* | 2 | 1 | *Claviceps purpurea (2)* |
| *Cokeromyces* | 1 | 1 | *Cokeromyces recurvatus (1)* |
| *Colletotrichum* | 2 | 2 | *Colletotrichum crassipes (1)- Colletotrichum musae (1)* |
| *Conidiobolus* | 1 | 1 | *Conidiobolus coronatus (1)* |
| *Cunninghamella* | 1 | 1 | *Cunninghamella bertholletiae (1)* |
| *Curvularia / Cochliobolus / Bipolaris* | 12 | 10 | *Curvularia affinis (1) -Curvularia geniculata (1) – Curvularia inaequalis (1) - Curvularia lunata (3) –Curvularia lunata var aeria (1) -Curvularia verruculosa (1) -Cochliobolus hawaiiensis (1) -Bipolaris sorokiniana (1) -Bipolaris spicifera (1) – Bipolaris victoriae (1)* |
| *Dicyma* | 1 | 1 | *Dicyma olivacea (1)* |
| *Dothideomyces* | 1 | 1 | *Dothideomyces sp. (1)* |
| *Emericellopsis* | 1 | 1 | *Emericellopsis synnematicola (1)* |
| *Emmonsia* | 3 | 3 | *Emmonsia parva (1) – Emmonsia parva var crescens (1) -Emmonsia pasteuriana (1)* |
| *Engyodontium* | 3 | 2 | *Engyodontium album (2) -Engyodontium parvisporum (1)* |
| *Epicoccum* | 4 | 1 | *Epicoccum nigrum (4)* |
| *Epidermophyton* | 3 | 1 | *Epidermophyton floccosum (3)* |
| *Eupenicillium* | 7 | 6 | *Eupenicillium cinnamopurpureum (1) -Eupenicillium javanicum (1) -Eupenicillium limoneum (1) -Eupenicillium pinetorum (1) -Eupenicillium shearii (2) -Eupenicillium terrenum (1)* |
| *Eutypella* | 3 | 1 | *Eutypella scoparia (3)* |
| *Exophiala* | 7 | 5 | *Exophiala alcalophila (1) -Exophiala dermatitidis (3) -Exophiala jeanselmei (1) -Exophiala salmonis (1) -Exophiala spinifera (1)* |
| *Exserohilum* | 1 | 1 | *Exserohilum rostratum (1)* |
| *Fennellomyces* | 1 | 1 | *Fennellomyces linderi (1)* |
| *Fissuricella* | 1 | 1 | *Fissuricella filamenta (1)* |
| *Fomitopsis* | 5 | 2 | *Fomitopsis palustris (2) – Fomitopsis pinicola (3)* |
| *Fonsecaea* | 1 | 1 | *Fonsecaea pedrosoi (1)* |
| *Fusarium /  Acremonium* | 166 | 49 | *F.acuminatum (1) -F.acutatum (8) -F.ananatum (6) -F.andiyazi (6) -F.annulatum (1) -F.anthophilum (5) -F.chlamydosporum (1) -F.delphinoides (2) -F.dimerum (1) -F.equiseti (3) -F.fujikuroi (7) -F.incarnatum (5) -F.lichenicola (2) -F.musae (2) -F.napiforme (8) -F.nygamai (7) -F.oxysporum (14) -F.oxysporum var redolens (1) -F.petroliphilum (3) –F.poae (1)- F..polyphialidicum (4) -F.proliferatum (13) -F.proliferatum var minus (1) -F.proliferatum var proliferatum (1) -F.sacchari (10) - F. sambucinum (1) - F.solani (7) -F.sporotrochioides (2) -F.subglutinans (3) -F.thapsinum (5) -F.trincinctum (1) -F.verticillioides (14) -A. af.pteridii (1) -A. breve (1) -A. butyri (1) -A. charticola (2) -A. chrysogenum (1) -A. falciforme (1) -A. furcatum (1) -A. fusidioides (1) -A. hansfordii (1) -A. implicatum (1) -A. kiliense (1) -A. longisporum (1) -A. ochraceum (1) -A. polychromum (1) -A. roseolum (1) -A. sclerotigenum (3) -A. strictum (1)* |
| *Galactomyces* | 5 | 2 | *Galactomyces geotrichum (3) -Geotrichum capitatum (2)* |
| *Ganoderma* | 1 | 1 | *Ganoderma resinaceum (1)* |
| *Geomyces* | 2 | 1 | *Geomyces pannorum (2)* |
| *Geosmithia* | 3 | 2 | *Geosmithia argillacea (1) -Geosmithia pallida (2)* |
| *Gilbertella* | 1 | 1 | *Gilbertella persicaria (1)* |
| *Gliocladium* | 1 | 1 | *Gliocladium viride (1)* |
| *Gliomastix* | 2 | 2 | *Gliomastix felina (1) -Gliomastix polychroma (1)* |
| *Glomerella* | 1 | 1 | *Glomerella cingulata (1)* |
| *Gymnascella* | 6 | 5 | *Gymnascella dankaliensis (2) -Gymnascella devroeyi (1) -Gymnascella hyalinospora (1) -Gymnascella marginispora (1) – Gymnascella udagawae (1)* |
| *Gymnoascus* | 2 | 2 | *Gymnoascus reessii (1) -Gymnoascus udagawae (1)* |
| *Hamigera* | 2 | 2 | *Hamigera fusca (1) -Hamigera insecticola (1)* |
| *Hansfordia* | 1 | 1 | *Hansfordia pulvinata (1)* |
| *Haplographium* | 1 | 1 | *Haplographium debellae-marengoi var equinum (1)* |
| *Hemicarpenteles* | 3 | 3 | *Hemicarpenteles acanthosporus (1) -Hemicarpenteles ornatus (1) -Hemicarpenteles paradoxus (1)* |
| *Hexagonia* | 1 | 1 | *Hexagonia hydnoides (1)* |
| *Histoplasma* | 1 | 1 | *Histoplasma capsulatum (1)* |
| *Hormoconis* | 1 | 1 | *Hormoconis resinae (1)* |
| *Hormographiella* | 1 | 1 | *Hormographiella verticilliata (1)* |
| *Hortaea* | 1 | 1 | *Hortaea werneckii (1)* |
| *Humicola* | 1 | 1 | *Humicola grisea var thermoidea (1)* |
| *Hyalodendron* | 1 | 1 | *Hyalodendron lignicola (1)* |
| *Hyphozyma* | 1 | 1 | *Hyphozyma variabilis (1)* |
| *Hypoxylon* | 2 | 2 | *Hypoxylon howeanum (1) -Hypoxylon lividipigmentum (1)* |
| *Isaria* | 2 | 1 | *Isaria farinosa (2)* |
| *Lecanicillium* | 2 | 2 | *Lecanicillium fungicola (1) -Lecanicillium psalliotae (1)* |
| *Lecythophora* | 1 | 1 | *Lecythophora sp. (1)* |
| *Macrophomina* | 1 | 1 | *Macrophomina phaseolina (1)* |
| *Madurella* | 1 | 1 | *Madurella pseudomycetomatis (1)* |
| *Malbranchea* | 1 | 1 | *Malbranchea arcuata (1)* |
| *Microsporum* | 39 | 15 | *M.audouinii (9) -M.boullardii (1) -M.canis (7) -M.canis var distortum (1) -M.cookei (2) -M.equinum (1) -M.ferrugineum (1) -M.fulvum (2) -M.gypseum (7) -M.nanum (1) -M.persicolor (2) -M.praecox (1) -M.racemosum (2) -M.rivalieri (1) -M.vanbreuseghemii (1)* |
| *Monodictys* | 1 | 1 | *Monodictys castanae (1)* |
| *Mucor* | 13 | 11 | *M. circinelloides (3) -M. durus (1) -M. ellipsoideus (1) -M. flavus (1) -M. fragilis (1) -M. indicus (1) -M. mucedo (1) -M. plumbeus (1) -M. racemosus f racemosus (1) -M. saturninus (1) -M. velutinosus (1)* |
| *Myceliophthora* | 2 | 2 | *Myceliophthora thermophila (1) -Myceliophthora vellerea (1)* |
| *Mycotypha* | 1 | 1 | *Mycotypha microsporum (1)* |
| *Myriodontium* | 2 | 1 | *Myriodontium keratinophilum (2)* |
| *Myrothecium* | 3 | 3 | *Myrothecium cinctum (1) -Myrothecium roridum (1) -Myrothecium verrucaria (1)* |
| *Myxotrichum* | 2 | 2 | *Myxotrichum chartarum (1) -Myxotrichum deflexum (1)* |
| *Neocosmospora* | 2 | 2 | *Neocosmospora vasinfecta (1) -Neocosmospora vasinfecta var africana (1)* |
| *Neofabraea* | 1 | 1 | *Neofabraea malicorticis (1)* |
| *Neoscytalidium* | 3 | 1 | *Neoscytalidium dimidiatum (3)* |
| *Neotestudina* | 1 | 1 | *Neotestudina rosatii (1)* |
| *Nigrospora* | 3 | 2 | *Nigrospora oryzae (1) – Nigrospora sphaerica (2)* |
| *Nodulisporium* | 4 | 3 | *Nodulisporium griseobrunneum (2) -Nodulisporium melonis (1) -Nodulisporium verrucosum (1)* |
| *Ochroconis* | 1 | 1 | *Ochroconis humicola (1)* |
| *Onychocola* | 1 | 1 | *Onychocola canadensis (1)* |
| *Ophidiomyces* | 1 | 1 | *Ophidiomyces ophiodiicola (1)* |
| *Ophiostoma* | 1 | 1 | *Ophiostoma stenoceras (1)* |
| *Paecilomyces* | 17 | 9 | *Paecilomyces amoeneroseus (1) -Paecilomyces carneus (1) –Paecilomyces formosus (1) - Paecilomyces inflatus (1) -Paecilomyces lilacinus (6) -Paecilomyces niphetodes (1) -Paecilomyces pascua (1) -Paecilomyces saturatus (1) – Paecilomyces variotii (4)* |
| *Parascedosporium* | 1 | 1 | *Parascedosporium putredinidis (1)* |
| *Penicillium / Talaromyces* | 141 | 94 | *P.af.ochrochloron (1) – P.af.toxicarum (1) – P.af.verruculosum (1) -P.allii (1) –P.allii-sativi (1) - P.asturianum (1) -P.atrosanguineum (2) -P.aurantiogriseum (1) –P.bilaiae (1) - P.brasilianum (2) –P.brevicompactum (2) - P.camemberti (2) -P.canescens (1) -P.capsulatum (1) -P.cecidicola (1) -P.cfr.simplicissimum (1) -P.chermesinum (2) -P.chrysogenum (6) -P.chrysogenum var dipodomyis (1) –P.citreonigrum (3) - P.citrinum (1) –P.copticola (1) - P.coralligerum (1) -P.corylophilum (2) -P.crateriforme (2) -P.crustosum (4) – P.decumbens (1) - P.digitatum (2) -P.expansum (2) -P.fellutanum (1) -P.funiculosum (2) -P.georgiense (2) -P.glabrum (4) -P.griseofulvum (3) -P.helicum (1) -P.hirsutum var albocoremium (1) -P.isariiforme (1) -P.janthinellium (1) -P.lividum (1) -P.loliense (1) -P.madriti (2) -P.mallochii (1) -P.mariae-crucis (2) -P.marneffei (2) -P.melanoconidium (1) -P.melinii (1) -P.minioluteum (1) -P.montanense (1) -P.nalgiovense (3) -P.neoechinulatum (1) -P.olsonii (2) -P.oxalicum (2) -P.pancosmium (1) -P.piceum (1) -P.pinophilum (2) -P.polonicum (2) -P.purpurescens (1) -P.purpurogenum (1) -P.raistrickii (1) – P.ramulosum (1) - P.resedanum (1) -P.restrictum (1) -P.rolfsii (2) -P.roqueforti (1) -P.rubens (2) -P.rugulosum (2) -P.sanguifluum (2) -P.sizovae (1) –P.smithii (2) - P.sp. (1) -P.spinulosum (2) -P.steckii (2) -P.striatisporum (1) -P.subrubescens (1) -P.terrigenum (1) -P.thomii (1) – P.toxicarum (1) - P.ulaiense (1) -P.variabile (1) -P.viridicatum (3) - Talaromyces allahabadensis (1) -Talaromyces amestolkiae (2) -Talaromyces coalescens (1) – Talaromyces diversus (1) – Talaromyces echinosporus (1) -Talaromyces flavovirens (1) – Talaromyces helicus (1) -Talaromyces leycettanus (1)- Talaromyces minioluteus (1) – Talaromyces pinophilus (1) – Talaromyces purpurogenus (1) – Talaromyces radicus (1) – Talaromyces siamensis (2) – Talaromyces stollii (2)* |
| *Pestalotiopsis* | 1 | 1 | *Pestalotiopsis hainanensis (1)* |
| *Phaeoacremonium* | 3 | 2 | *Phaeoacremonium parasiticum (2) -Phaeoacremonium scolyti (1)* |
| *Phaeotheca* | 1 | 1 | *Phaeotheca triangularis (1)* |
| *Phanerochaete* | 2 | 1 | *Phanerochaete chrysosporium (2)* |
| *Phialemonium* | 1 | 1 | *Phialemonium globosum (1)* |
| *Phialophora* | 3 | 3 | *Phialophora bubakii (1) -Phialophora japonica (1)- Phialophora olivacea (1)* |
| *Phlebia* | 5 | 3 | *Phlebia acerina (1) – Phlebia radiate (1) – Phlebia tremellosa (3)* |
| *Phoma* | 2 | 2 | *Phoma macrostoma var macrostoma (1) -Phoma sp (1)* |
| *Phycomyces* | 1 | 1 | *Phycomyces blakesleeanus (1)* |
| *Pithomyces* | 2 | 1 | *Pithomyces chartarum (2)* |
| *Pleospora* | 1 | 1 | *Pleospora herbarum (1)* |
| *Pleurostomophora* | 1 | 1 | *Pleurostomophora richardsiae (1)* |
| *Pochonia* | 1 | 1 | *Pochonia bulbillosa (1)* |
| *Porostereum* | 1 | 1 | *Porostereum spadiceum (1)* |
| *Pseudallescheria / Scedosporium* | 20 | 8 | *P.angusta (2) -P.apiosperma (5) -P.boydii (3) -P.desertorum (1) -P.ellipsoidea (2) -P.minutispora (2) -S.aurantiacum (1) - S.prolificans (4)* |
| *Pseudeurotium* | 2 | 2 | *Pseudeurotium bakeri (1) –Pseudeurotium zonatum (1)* |
| *Pseudomicrodochium* | 1 | 1 | *Pseudomicrodochium fusarioides (1)* |
| *Pseudozyma* | 2 | 2 | *Pseudozyma aphidis (1) -Pseudozyma rugulosa (1)* |
| *Radulidium* | 1 | 1 | *Radulidium subulatum (1)* |
| *Rhizomucor* | 7 | 3 | *Rhizomucor miehei (1) -Rhizomucor pusillus (5) -Rhizomucor variabilis (1)* |
| *Rhizopus* | 12 | 7 | *Rhizopus homothallicus (1) -Rhizopus microsporus rhizopodiformis (2) -Rhizopus oryzae (5) -Rhizopus schipperae (1) -Rhizopus sexualis (1) -Rhizopus stolonifer (1) –R. stolonifer var stolonifer (1)* |
| *Sagenomella* | 1 | 1 | *Sagenomella diversispora (1)* |
| *Schizophyllum* | 3 | 1 | *Schizophyllum commune (3)* |
| *Scopulariopsis* | 12 | 9 | *Scopulariopsis acremonium (1) -Scopulariopsis asperula (1) -Scopulariopsis atra (1) -Scopulariopsis brevicaulis (4) – Scopulariopsis brumptii (1) - Scopulariopsis candida (1) -Scopulariopsis cinerea (1) -Scopulariopsis fusca (1) – Scopulariopsis murina (1)* |
| *Scytalidium* | 3 | 3 | *Scytalidium dimidiatum (1) -Scytalidium hyalinum (1) -Scytalidium lignicola (1)* |
| *Septofusidium* | 1 | 1 | *Septofusidium berolinense (1)* |
| *Sordaria* | 1 | 1 | *Sordaria fimicola (1)* |
| *Spicellum* | 1 | 1 | *Spicellum roseum (1)* |
| *Sporothrix* | 3 | 3 | *Sporothrix inflata (1) – Sporothrix insectorum (1) -Sporothrix schenckii (1)* |
| *Sporotrichum* | 3 | 2 | *Sporotrichum aurantiacum (1) -Sporotrichum pruinosum (2)* |
| *Stachybotrys* | 1 | 1 | *Stachybotrys chartarum (1)* |
| *Syncephalastrum* | 3 | 1 | *Syncephalastrum racemosum (3)* |
| *Thanatephorus* | 7 | 1 | *Thanatephorus cucumeris (7)* |
| *Thielavia* | 1 | 1 | *Thielavia heterothallica (1)* |
| *Tilletiopsis* | 1 | 1 | *Tilletiopsis minor (1)* |
| *Trametes* | 2 | 2 | *Trametes trogii (1) – Trametes versicolor (1)* |
| *Trichoderma /  Hypocrea* | 23 | 17 | *T.aureoviride (1) – T.citrinoviride (2) -T.ghanense (1) -T.harzianum (2) -T.koningii (1) -T.koningiopsis (1) -T.longibrachiatum (5) -T.parceramosum (1) -T.polysporum (1) -T.virens (1) -T.viride (1) -T.viridescens (1) -H.atrogelatinosa (1) -H.hunua (1) -H.parapilulifera (1) -H.schweinitzii (1) -H.vinosa (1)* |
| *Trichophyton /  Arthroderma* | 115 | 39 | *T.ajelloi (3) -T.concentricum (1) -T.eboreum (1) - T.indicum (1) -T.interdigitale (9) –T.kuryangei (3) - T.mentagrophytes (9) -T.mentagrophytes var batonrougei (1) -T.mentagrophytes var erinacei (2) -T.mentagrophytes var porcellae (3) -T.persicolor (1) -T.phaseoliforme (1) -T.quinckeanum (3) -T.rubrum (12) -T.schoenleinii (3) - T.soudanense (4) -T.terrestre (2) -T.tonsurans (6) -T.vanbreuseghemii (1) -T.verrucosum (1) -T.violaceum (5) -A.benhamiae (7) -A.borellii (1) -A.cookiellum (2) -A.corniculatum (2) -A.crocatum (1) -A.cuniculi (2) -A.curreyi (2) -A.fulvum (3) -A.gloriae (1) -A.grubyi (1) -A.gypseum (3) -A.incurvatum (2) -A.lenticulare (2) -A.multifidum (2) -A.persicolor (2) -A.racemosum (2) -A.simii (3) – A.vanbreuseghemii (5)* |
| *Trichosporiella* | 2 | 2 | *Trichosporiella cerebriformis (1) -Trichosporiella ornithopoda (1)* |
| *Trichothecium* | 2 | 1 | *Trichothecium roseum (2)* |
| *Tyromyces* | 1 | 1 | *Tyromyces fissilis (1)* |
| *Ulocladium* | 1 | 1 | *Ulocladium oudemansii (1)* |
| *Ustilago* | 4 | 1 | *Ustilago cynodontis (4)* |
| *Venturia* | 1 | 1 | *Venturia inaequalis (1)* |
| *Verticillium* | 1 | 1 | *Verticillium tenerum (1)* |
| *Westerdykella* | 1 | 1 | *Westerdykella sp. (1)* |
| *Wolfiporia* | 1 | 1 | *Wolfiporia cocos (1)* |
| *Ybotromyces* | 1 | 1 | *Ybotromyces caespitosus (1)* |
